# Efficacy of low-level laser therapy for subacromial impingement syndrome: a meta-analysis of randomized controlled trials

**DOI:** 10.1007/s10103-026-05028-7

**Published:** 2026-09-14

**Authors:** Liangsheng Chen, Yage Wang

**Affiliations:** 1 Department of Rehabilitation Medicine, Henan Provincial People’s Hospital, Zhengzhou, China; 2 School of Rehabilitation Medicine, Henan University of Chinese Medicine, Zhengzhou, China

**Keywords:** Subacromial impingement syndrome, Low-level laser therapy, Photobiomodulation, Shoulder pain

## Abstract

To systematically explore the therapeutic effects of low-level laser therapy (LLLT) on pain, shoulder range of motion (ROM), and functional disability in patients with subacromial impingement syndrome (SIS). Eight databases were systematically searched. We assessed study quality with the Cochrane Risk of Bias tool, and evidence quality was evaluated using the GRADE approach. Seventeen studies and twenty-one RCTs were included, with 1118 patients enrolled. Relative to the control group, LLLT markedly reduced pain (MD = -0.54, 95% CI: -1.05 to -0.02, *P* = 0.04). LLLT also significantly improved shoulder flexion (MD = 4.35, 95% CI: 1.13 to 7.58, *P* = 0.008), abduction (MD = 3.72, 95% CI: 0.79 to 6.64, *P* = 0.01), and external rotation (MD = 1.96, 95% CI: 0.51 to 3.42, *P* = 0.008). However, no significant improvement was found in shoulder extension (MD = 0.79, 95% CI: -0.47 to 2.06, *P* = 0.22), internal rotation (MD = 1.14, 95% CI: -1.51 to 3.78, *P* = 0.40), and functional disability (MD = -3.61, 95% CI: -9.49 to 2.26, *P* = 0.23). Low-quality evidence suggests that LLLT effectively alleviates pain in patients with SIS and enhances shoulder flexion, abduction, and external rotation ROM. Very low-quality evidence indicates that LLLT exerts no significant effect on shoulder functional disability, extension, and internal rotation ROM. Additionally, we suggest that clinicians reassess the need for continued treatment when the number of sessions approaches 12, rather than routinely adopting extended protocols. Owing to the high heterogeneity among the included studies, the above conclusions are regarded as exploratory findings.

**Supplementary Information:**

The online version contains supplementary material available at https://doi.org/10.1007/s10103-026-05028-7.

## Introduction

Shoulder pain is the third most common disorder of the musculoskeletal system, significantly compromising patients’ ability to work and their overall quality of life [1]. Among all shoulder pain cases, 44%–65% stems from subacromial impingement syndrome (SIS) [2]. SIS is a clinical spectrum of disorders caused by impingement between the humeral head and acromion, leading to injury to periarticular shoulder structures [3, 4]. Its incidence increases with advancing age and it predominantly affects individuals around 60 years old [5]. Beyond pain, it also causes restricted shoulder range of motion (ROM), which impairs patients’ daily self-care activities such as combing hair and dressing. In severe cases, it can disturb patients’ sleep and even cause anxiety [6, 7].

Clinicians give priority to conservative management for 3 months and only consider surgery when the treatment response is suboptimal [6]. Common conservative treatments mainly include physical therapy, such as shockwave therapy, and low-level laser therapy (LLLT) [8]. LLLT induces intracellular biochemical reactions and is therefore also referred to as photobiomodulation; it acts directly on nociceptive nerve endings and inhibits the transmission of pain signals to center, thereby exerting analgesic effects. Several randomized controlled trials (RCTs) have confirmed that LLLT could effectively relieve pain in various musculoskeletal conditions [9].

To date, only the meta-analyses by El Tayeb et al. [10] and Giulia et al. [8] have explored the therapeutic effects of LLLT on SIS. However, these two studies have several limitations. First, the number of included RCTs was limited (2 and 10 studies, respectively), and their statistical power was insufficient to detect small-to-moderate effect sizes. Second, the outcomes were incomplete, with ROM limited to a single direction. Third, the two meta-analyses reached inconsistent conclusions regarding whether LLLT is superior to other conservative treatments, and neither examined key factors that may influence treatment efficacy, such as treatment dose and number of sessions. Therefore, the precise therapeutic effects of LLLT on SIS and its optimal treatment parameters remain unclear.

To address these gaps, we incorporated the latest RCT evidence to systematically evaluate the effects of LLLT on pain, multiplanar shoulder ROM, and shoulder function in patients with SIS. We also performed subgroup analyses to explore potential factors influencing treatment efficacy, aiming to provide more comprehensive evidence for clinical decision-making.

## Methods

The study was registered in the PROSPERO (CRD420261380753), and the study protocol was strictly implemented in accordance with the PRISMA checklist (see Additional file 4).

### Retrieval strategy

Chen and Wang performed searches in eight databases: PubMed, Web of Science, Embase, Cochrane Library, CNKI, Wanfang, CBM, and VIP. Meanwhile, they also searched relevant reviews to avoid missing relevant literature (Deadline: April 29, 2026). Search strategy outline as follows: (“Shoulder Impingement Syndrome” OR “Rotator Cuff Impingement Syndrome” OR “Shoulder Impingement”) AND (“Laser Therapy” OR “Laser Scalpels” OR “Therapies, Laser”). The complete search strategy is in Additional file 1.

### Inclusion and exclusion criteria

Inclusion criteria:

- P (Population): Patients diagnosed with SIS through clinical and/or imaging assessments, with no restrictions on age or gender.
- I (Intervention): LLLT, administered alone or combined with other conservative treatments.
- C (Comparison): Sham laser therapy, exercise therapy, therapeutic ultrasound, kinesiology taping (KT).
- , or other physical therapies.
- O (Outcome): Report at least one of the outcomes: Visual Analogue Scale (VAS) pain score [11], Shoulder Pain and Disability Index (SPADI) [12], or shoulder ROM [13].
- S (Study design): RCTs.

Exclusion criteria: (1) Conference abstracts, study protocols, and reviews; (2) Animal studies; (3) Studies focusing on high-intensity laser therapy (HILT); (4) Clinical studies on shoulder pain in which participants did not meet the diagnostic criteria for SIS; (5) Non-RCTs; (6) Publications with unavailable full texts or incomplete data.

### Data extraction

Chen and Wang extracted key data: first author; publication year; country; sample; age; gender; intervention and control protocols; LLLT treatment parameters (device, wavelength, output power, irradiation frequency, single-point irradiation area, cumulative energy per point, duration of single irradiation, total number of interventions, and treatment courses); mean and standard deviation (SD). If discrepancies emerged between the two investigators, a consensus was reached through discussion.

The primary outcomes of this analysis included pain, shoulder function, and shoulder ROM. Pain was evaluated via VAS, while shoulder function was measured using SPADI. All outcomes were assessed at baseline and post-intervention. Regarding ROM measures, we extracted active range of motion (AROM) preferentially when both AROM and passive range of motion (PROM) were reported in the included studies. In cases where only one type of ROM was reported, we extracted that measure accordingly. This approach was adopted to ensure consistency across studies while maximizing the use of available data. For the primary analysis, we extracted data from the earliest post-intervention time point in each trial. Follow-up data were incorporated into subgroup analyses. If a single trial reported multiple follow-up time points, we prioritized data recorded at the predefined primary outcome time point set by the original authors.

For studies reporting only median, range, or interquartile range (IQR), we adopted the conversion method proposed by Wan et al. to derive the corresponding mean and SD [14]. Second, for studies with multiple groups, the data of the shared group were kept unchanged to avoid duplicate inclusion, while only its sample size was equally divided. In this study, Alfredo et al. [15], Calis et al. [16], Sen et al. [17], and Agir et al. [18] reported trials with multiple groups, which were processed using the above method.

### Data analysis

Data analysis was conducted using RevMan 5.4 and Stata 18.0. Uniform assessment scales were used for all outcomes, and effect sizes were calculated as mean difference (MD) with 95% confidence interval (CI). All outcomes were analyzed using the random effects model [19]. Additionally, between-study heterogeneity was represented by I². We also assessed publication bias via funnel plots and Egger’s test [20]. Sensitivity analysis was performed to verify the robustness of the pooled results. In addition, we conducted subgroup analyses and meta-regression with follow-up duration, intervention frequency, intervention types and total number of interventions (Both analyses adopted the statistical threshold of *P* < 0.05.). Meta-regression was performed using a mixed-effects model with restricted maximum likelihood (REML) estimation. All variables, including treatment sessions and follow-up duration, were entered as continuous variables. Except for intervention type, the other two variables (treatment sessions and follow-up duration) were treated as continuous.

### Quality evaluation

The methodological quality was assessed with the Cochrane Risk of Bias [21]. The quality of evidence was rated using the GRADE system [22]. All assessments were performed by Chen and Wang.

## Results

### Literature screening process

**Fig. 1:**
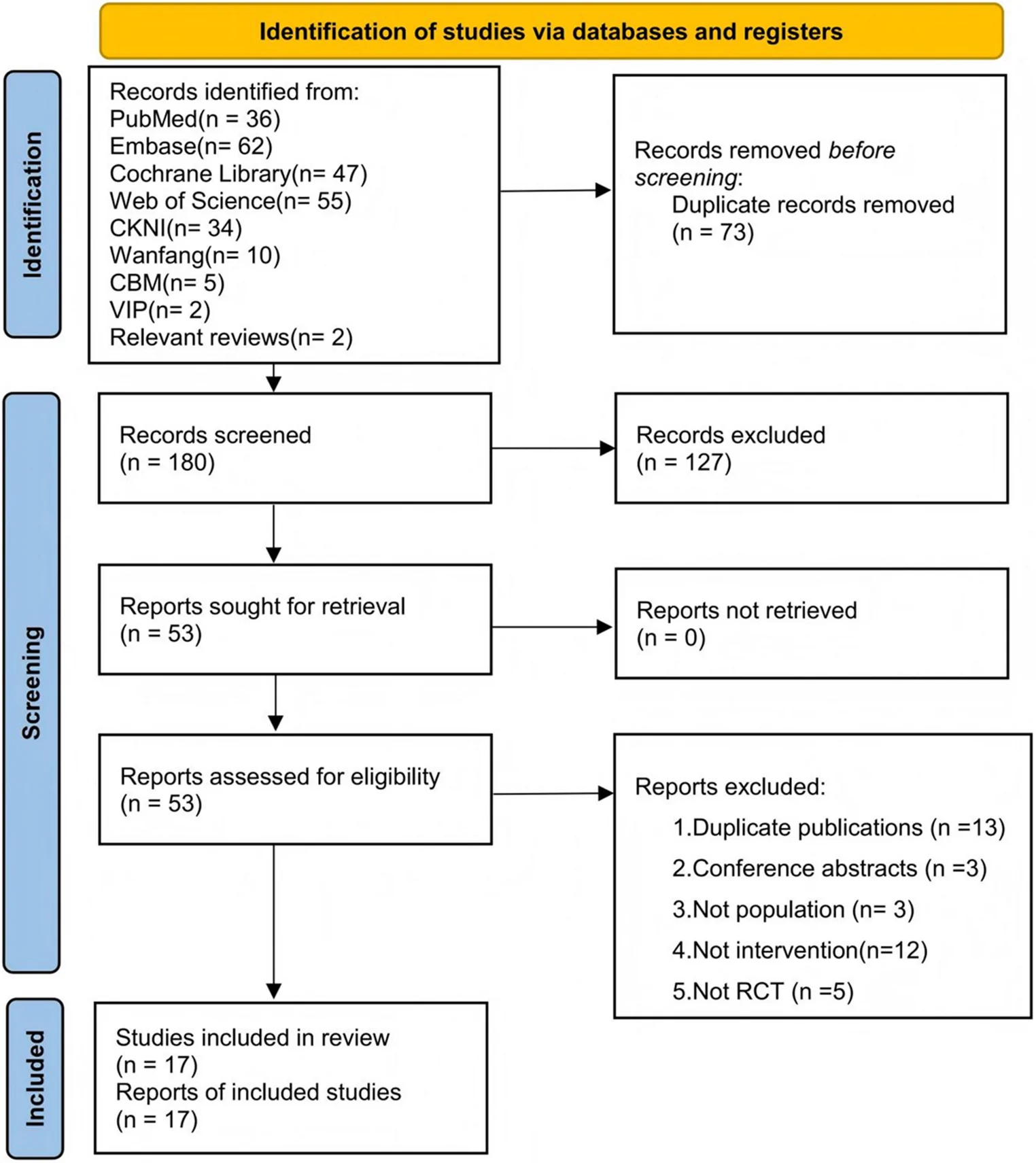
Flow diagram of study selection

Chen and Wang identified 253 records from eight databases. They initially eliminated 73 duplicates and excluded another 127 records after reviewing titles and abstracts. Of the remaining 53 studies, 13 duplicate publications, 3 conference abstracts, 12 HILT-related studies, 3 general shoulder pain studies, and 5 non-RCTs were excluded due to ineligibility. The remaining 15 studies were included. Besides, two additional studies were included by screening relevant reviews. Ultimately, 17 studies were included [15–19, 24–36] (Fig. 1).

### Study characteristics

These studies were conducted in five countries across three continents. LLLT sessions varied from 7 to 24, and the intervention duration spanned 2 to 8 weeks (Table 1). Regarding LLLT treatment parameters, 15 studies reported single intervention duration (1–10 min) [15–19, 24–27, 29–31, 33–36]; 11 studies reported single-point irradiation time (20–150 s) [15, 17, 18, 24–29, 33, 34]; All studies reported laser wavelength (820–1064 nm); 6 studies reported irradiation frequency (16 Hz–3 MHz) [15, 16, 18, 23, 24, 27]; 11 studies reported single-point irradiation area (0.07–20 cm²) [15, 18, 24–26, 29, 32–36]; 14 studies reported cumulative energy per point (1–20 J) [15–19, 24–29, 33–36]. Detailed treatment parameters are shown in Table 2.

For the experimental groups: 7 studies used LLLT combined with exercise therapy or KT [15, 18, 26, 27, 31, 32, 35]; 2 studies used LLLT alone [28, 33]; 8 studies used LLLT combined with exercise therapy and other physical therapies [16, 17, 24–26, 29, 30, 34]. For the control groups: 5 studies used sham laser combined with exercise therapy or KT [25–27, 29, 31]; 4 studies used physical therapy alone [15, 18, 27, 32]; 7studies used exercise therapy combined with other physical therapies [17–19, 23, 30, 34, 35].

**Table 1:** Baseline characteristics of included studies

Author (year)	Country	Sample	Age	Gender	Interventions	Sessions	Outcome
Bal [24]	Turkey	40	EG: 51.7 ± 14.1	EG: F (15), M (5)	EG: LLLT+exercise + hot pack+cold pack	T0: baseline T1: 2nd week T2: 12th week	VAS SPADI
			CG: 53.1 ± 8.4	CG: F (13), M (7)	CG: Exercise + hot pack+cold pack		
Yeldan [25]	Turkey	60	EG: 55.32	EG: F (25), M (9)	EG: LLLT+exercise+cold	T0: baseline T1: 3rd week No follow-up	VAS ROM
			CG: 55.00	CG: F (22), M (4)	CG: Placebo laser+exercise+cold		
Dogan [26]	Turkey	52	EG: 53.7 ± 12.6	EG: F (20), M (10)	EG: LLLT+exercise+cold	T0: baseline T1: post-treatment, 14th day No follow-up	VAS ROM SPADI
			CG: 53.45 ± 9.64	CG: F (13), M (9)	CG: Placebo laser+exercise+cold		
Abrisham [27]	Iran	80	EG: 52.2 ± 5.7	EG: F (24), M (16)	EG: LLLT+exercise	T0: baseline T1: post-treatment, 2nd week No follow-up	VAS ROM
			CG: 51.2 ± 6.7	CG: F (26), M (14)	CG: Placebo laser+exercise		
Calis [17]	Turkey	52	EG: 46.2 ± 12.14	EG: F (10), M (5)	EG: LLLT+exercise + hot pack	T0: baseline T1: 15th day No follow-up	VAS
			CG1:50.34 ± 13.69; CG2:50.42 ± 12.41	CG1:F (11), M (5); CG2:F(14),M(7)	CG1: Hot pack+exercise; CG2: Ultrasound+exercise + hot pack		ROM
Somashekhar [36]	India	60	EG: 27.60 ± 1.72	EG: F (13), M (17)	EG: Laser+exercise	T0: baseline T1:3rd day T2: 6th day T3: 9th day T4: 12th day T5: 15th day No follow-up	VAS ROM
			PG: 28.13 ± 1.41	CG: F (15), M (15)	CG: Ultrasound+exercise		
Eslamian 2011	Iran	50	EG: 50.16 ± 12.10	EG: F (10), M (15)	EG: LLLT+exercise + hot pack+ultrasound+TENS	T0: baseline T1: 3rd week	VAS ROM
			PG: 50.28 ± 11.72	CG: F (16), M (9)	CG: Exercise + hot pack+ultrasound+TENS		
Abd-Allah 2017	Egypt	30	EG: 42.46 ± 6.13	EG: F (8), M (7)	EG: LLLT+Exercise	T0: baseline T1: post-treatment, 12th day No follow-up	VAS
			CG: 41.46 ± 6.97	CG: F (11), M (4)	CG: Exercise		
Alfredo 2020	Brazil	120	EG: 51.9 ± 8.7	EG: (*n* = 42)	EG: LLLT+Exercise	T0: baseline T1: 8weeks Follow-up: 3rd months	SPADI
			CG: 56.0 ± 10.4	CG: (*n* = 42)	CG: Exercise		
Ozmen [29]	Turkey	120	EG: 50.78 ± 11.22	EG: F (41), M (19)	EG: LLLT	T0: baseline T1: post-treatment No follow-up	VAS
			CG: 50.42 ± 11.41	CG: F (40), M (20)	CG: Isokinetic		
Actici 2015	Turkey	60	EG: 51.3 ± 8.7	EG: F (22), M (8)	EG: LLLT+Exercise+TENS + hot pack	T0: baseline T1: post-treatment No follow-up	VAS ROM
			CG: 54.5 ± 7.2	CG: F (23), M (7)	CG: Placebo laser+exercise+TENS + hot pack		
Ravish [31]	India	60	18–60	EG: F (14), M (16)	EG: LLLT+exercise+therapeutic Kinesio taping	T0: baseline T1: post-treatment	VAS SPADI ROM
				CG: F (12), M (18)	CG: Ultrasound+exercise+therapeutic Kinesio taping		
Badıl 2020	Turkey	64	EG: 48(34–65)	EG: F (24), M (10)	EG: LLLT	T0: baseline T1: post-treatment Follow-up: 3rd month	VAS ROM SPADI
			CG: 49(36–65)	CG: F (14), M (16)	CG: Ultrasound		
Zaki [32]	Iran	20	EG:51.5 ± 9.26	EG: F (6), M (4)	EG: LLLT+therapeutic Kinesio taping	T0: baseline T1: post-treatment No follow-up	VAS SPADI
			CG:53.43 ± 11.4	CG: F (6), M (4)	CG: Placebo laser+therapeutic Kinesio taping		
Sen [18]	Turkey	54	EG:52.2 ± 8.3	EG: F (15), M (4)	EG: LLLT+cold-pack therapy + HBE	T0: baseline T1: 1 st month T2:3rd month	VAS SPADI
			CG1:54.7 ± 10.7; CG2:48.5 ± 11.9	CG1: F (13), M (4); CG2:F (10), M (8)	CG1:Cold-pack therapy + HBE; CG2:Ultrasound+cold-pack therapy + HBE		
Agir [19]	Turkey	168	EG:51.48 ± 5.51	EG: F (47), M (9)	EG: LLLT+exercise	T0: baseline T1: post-treatment T2: 1 st month	VAS SPADI ROM
			CG1:47.61 ± 10.49	CG1: F (48), M (8)	CG1: Exercise		
			CG2:49.05 ± 10.30	CG2:F(49),M(7)	CG2:Peloids therapy+exercise		
Saleh [33]	Egypt	28	EG:38.1 ± 5.95	EG: F (9), M (5)	EG: LLLT+exercise	T0: baseline T1: post-treatment No follow-up	SPADI ROM
			CG:37.64 ± 5.26	CG: F (11), M (3)	CG: Exercise		

Age is presented as mean ± SD. *EG* experimental group, *CG* control group, *F* female, *M* male, *VAS* visual analogue scale, *SPADI* shoulder pain and disability index, *ROM* range of motion, *LLLT* low-level laser therapy, *TENS* transcutaneous electrical nerve stimulation, *HBE* home-based exercise

**Table 2:** LLLT intervention parameters

Study	Total sessions	Site	Device	Specific parameters					
				Dur(min)	Wav (nm)	Frequency (HZ)	Spot size (cm^2^)	Cum(J)	Per (s)
Bal [24]	5times/wk×2wk	The tuberculum majus and minus, the anterior and posterior faces of the capsule, and the subacromial regions.	A Ga-As diode laser instrument (Roland Serie, Elettronica Pagani, Paderno, Italy, Mod IR 27/1)	10	904	5500	0.8	1.6	120
Yeldan [25]	5times/wk×3wk	The subacromial and anterior shoulder regions	GaAs diode laser instrument (Roland Serie Elettronica Pagani)	8	904	2000	15	3	90
Dogan [26]	5times/wk×2.8wk	The subacromial region of the shoulder	The Gallium-Aluminum-Arsenide (GaAlAs, infrared laser)	5–6	850	NR	0.07	5	60
Somashekhar [36]	5times/wk×2wk	NR	NR	5–6	850	NR	0.07	5	NR
Eslamian 2011	10times	Over 10 shoulder tender points	The gallium-aluminum-arsenide (Ga-Al-As) infrared diode laser 476	5	830	NR	1	4	20
Alfredo 2020	3times/wk×8wk	The supraspinatus muscle tendon, on the subacromial bursa, and along the bicipital groove.	The Irradia Class 3B (Stockholm, Sweden)	2.5	904	700	0.5	3	50
Ozmen [29]	7times/wk×2wk	Omuz çevresinde tespit edilen 8 ayrı noktaya	Chattanooga Kombine Elektroterapi cihazı and diyot lazer	8	830	NR	NR	6	NR
Actici 2015	5times/wk×3wk	The subacro- mial space, supraspinatus tendon insertion, and gle- nohumeral joint	A gal- lium-aluminum-arsenide (GaAlAs, infrared laser) diode laser device (Chattanooga Group, USA)	2	850	NR	0.07	4	40
Ravish [31]	3times/wk×3wk	NR	low level laser	3	820	NR	NR	NR	NR
Badıl 2020	5times/wk×3wk	The subacromial area	NR	5	1064	NR	0.12	7	60
Sen [18]	5times/wk×3wk	The greater and lesser tubercles, the bicipital groove, and the anterior *and posterior* aspects of the capsule	Gallium- aluminum-arsenide diode laser device (Chattanooga Medical Supply Inc., TN, USA)	5	850	NR	NR	3	60
Agir [19]	5times/wk×2wk	The subacromial space, glenohu- meral space, and adhesion site of the supraspinatus tendon	The gallium- aluminium-arsenide (GaAIAs) diode laser (Chattanooga, Mexico, USA)	2	860	1000	15–20	3.6	40
Abrisham [27]	5times/wk×2wk	glenohumeral joint, Rotator cuff tendon, glenohumeral joint	A Ga As laser device (Model Laserpet 100, Petas Co., Turkey)	6	890	NR	NR	2–4	120
Cails 2011	15consecutive days	Shouder	A Ga As laser device (Model Laserpet 100, Petas Co., Turkey)	2	904	16	NR	1	NR
Abd-Allah 2017	3times/wk×4wk	Shoulder behind the patient back	GaAs LASER (IR) (Phyaction 740, Gymna Unify N.V., Belgium)	NR	905	3 M	NR	NR	150
Zaki [32]	3times/wk×2.33wk	subacromial space and rotator cuff tendons	The Diode laser (OptonPro, Zimmer MedizinSysteme GmbH, Junkersstraße 9, 89,231 Neu- Ulm, Bavaria, Southern Germany)	NR	810	NR	0.8	NR	NR
Saleh [33]	3times/wk×3wk	The supraspinatus muscle tendon, the subacromial bursa, and the bicipital groove	The diode laser device (Chattanooga Group, USA, model 27841)	1.67	850	NR	1	20	NR

*NR* no report, *wk* week

### Quality evaluation results

**Fig. 2:**
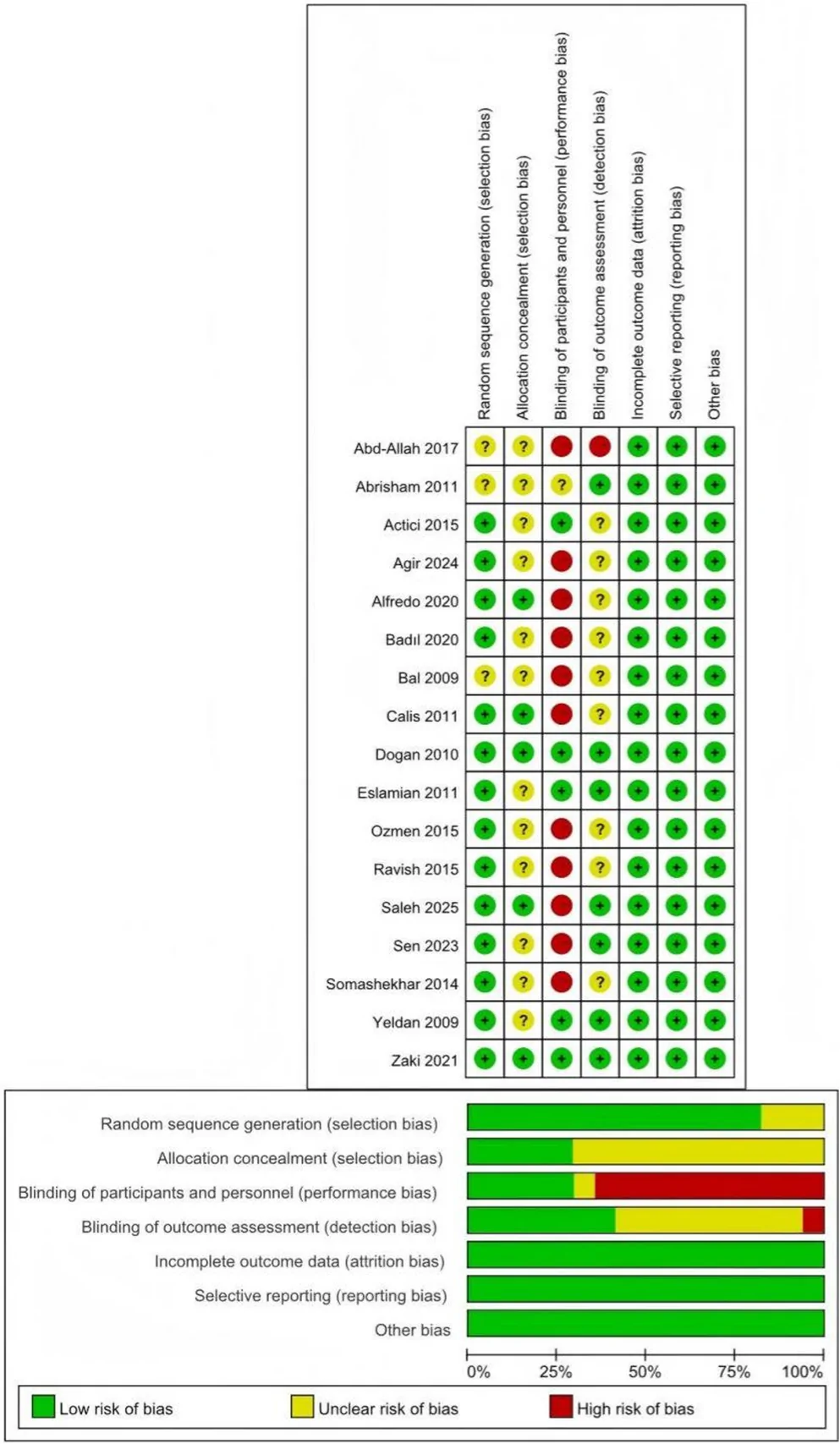
Risk of bias assessment table

For random sequence generation, 14 (82%) studies were rated as low risk of bias. With regard to allocation concealment, only 5 (29%) studies were judged as low risk. For blinding of participants and personnel, merely 5 (29%) studies were classified as low risk. For blinding of outcome assessment, 7 (41%) studies were judged as low risk. Seventeen (100%) studies were rated as low risk with regard to incomplete outcome data, selective outcome reporting, and other sources of bias (see Fig. 2)

Overall, the methodological quality of the included studies varied substantially. Performance was satisfactory in random sequence generation and completeness of outcome data. By contrast, blinding of participants and personnel was generally poor, with 65% rated as high risk, and allocation concealment remained largely unclear (71% judged as unclear risk). The overall blinding methodology was inadequate, which may compromise the measurement of subjective outcomes such as pain and reduce the reliability of pooled data.

### Meta-analysis results

#### Pain

**Fig. 3:**
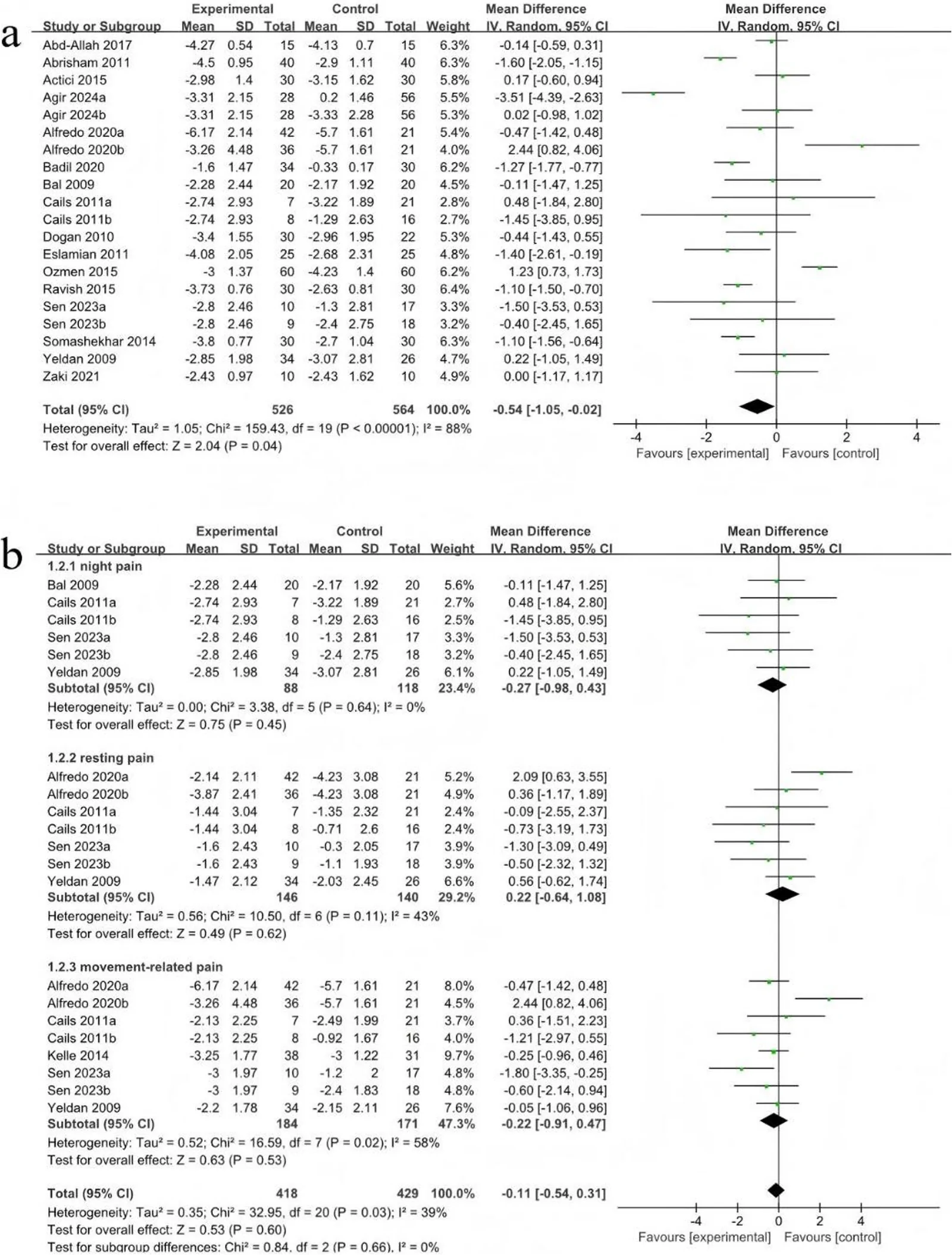
(**a**) Forest plot for pain outcome (LLLT vs. control); (**b**) Forest plot for pain characteristics (night pain, movement-related pain and resting pain)

Sixteen studies involving 20 RCTs with 1090 participants were analyzed for this outcome. Meta-analysis revealed that LLLT yielded substantial pain relief in patients with SIS (MD = −0.54, 95% CI: −1.05 to −0.02, *P* = 0.04), with substantial heterogeneity across studies (I² = 88%) (Fig. 3a). Subgroup analyses did not identify the source of heterogeneity (see Additional file 2). This finding suggested that the high heterogeneity might have arisen from differences in LLLT parameter settings and varied intervention strategies across studies, which could not be explained by the predefined subgroup variables. Subgroup analysis stratified by pain characteristics showed that LLLT produced comparable analgesic effects on night pain, movement-related pain and resting pain, with no significant differences in therapeutic efficacy among subgroups (Fig. 3b).

#### Shoulder pain and disability

**Fig. 4:**
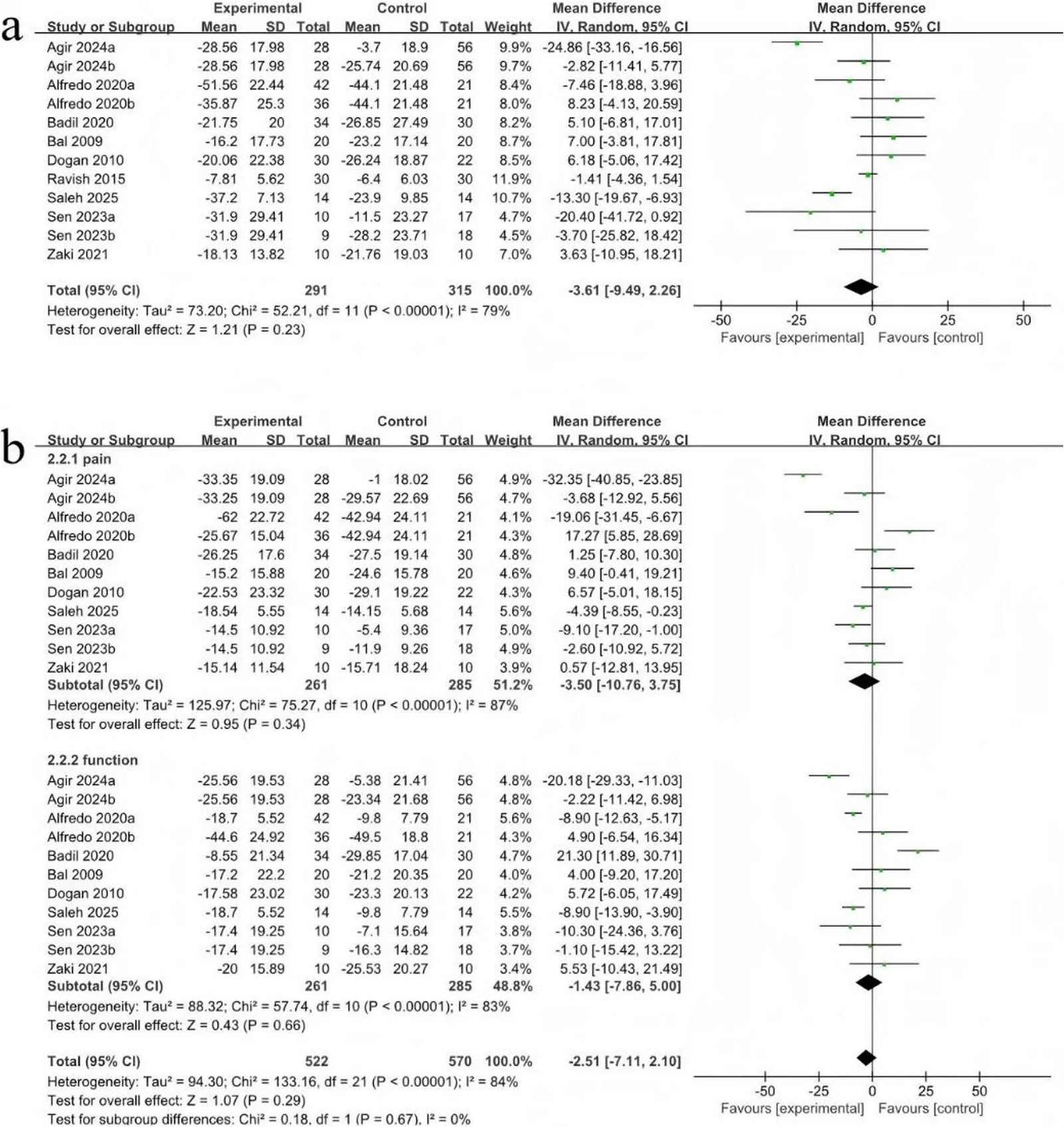
(**a**) Forest plot for shoulder pain and disability (LLLT vs. control); (**b**) Forest plot for shoulder pain and disability (SPADI subscale)

A total of 10 studies involving 12 RCTs with 606 participants were included for this outcome. LLLT did not yield significant improvements in SPADI scores (MD = −3.61, 95% CI: −9.49 to 2.26, *P* = 0.23), with substantial heterogeneity across the studies (I² = 79%) (Fig. 4a). Subgroup analysis revealed that in strata based on experimental intervention types, the subgroup receiving LLLT combined with exercise therapy or KT reached statistical significance in SPADI score improvement, while the LLLT alone subgroup showed no significant effect. Furthermore, neither the SPADI pain subscale nor function subscale scores differed significantly from those in the control group (Fig. 4b).

#### ROM of the shoulder joint

1. Flexion

**Fig. 5:**
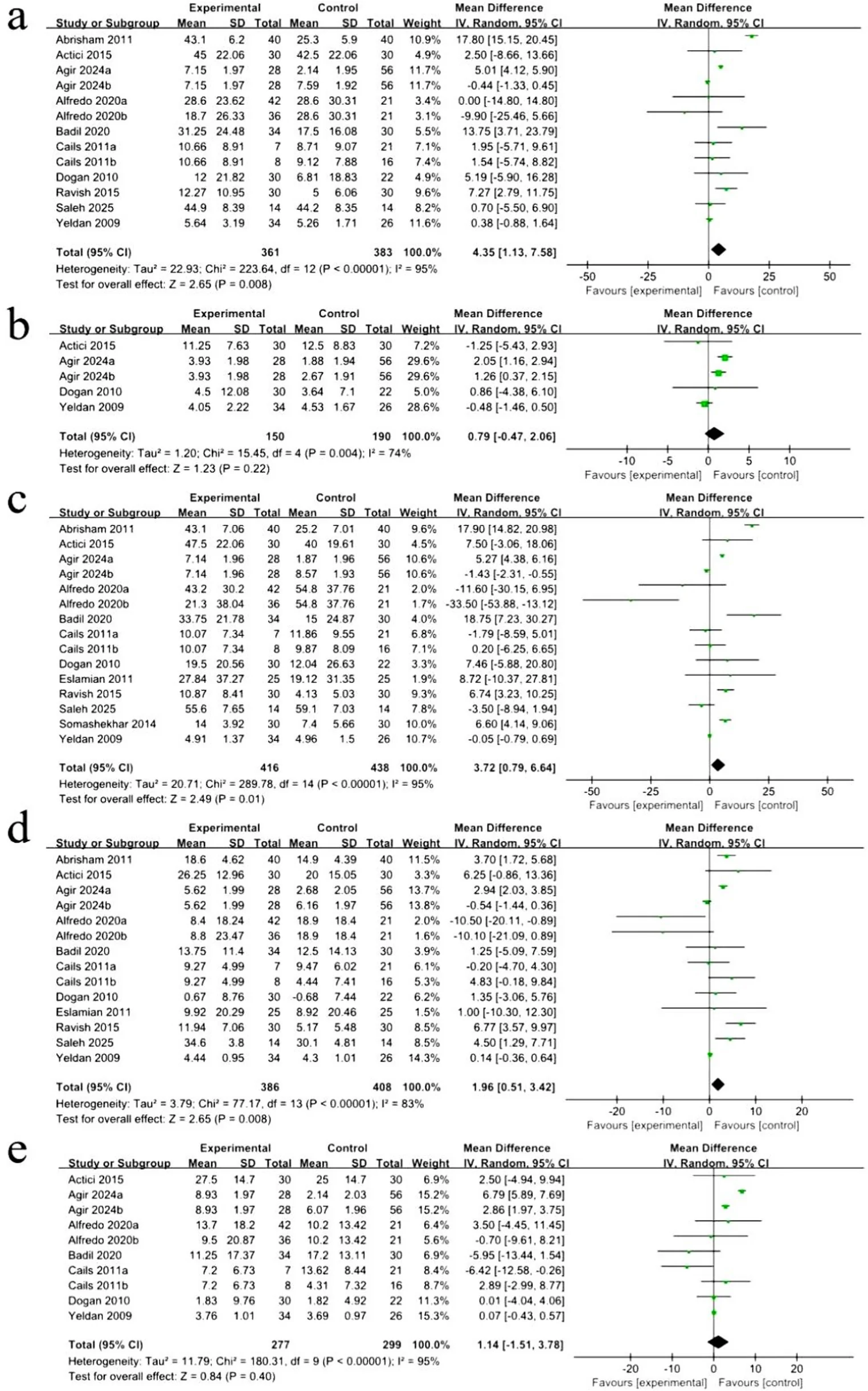
(**a**) Forest plot for shoulder flexion (LLLT vs. control); (**b**) Forest plot for shoulder extension (LLLT vs. control); (**c**) Forest plot for shoulder abduction (LLLT vs. control); (**d**) Forest plot for shoulder external rotation (LLLT vs. control); (**e**) Forest plot for shoulder internal rotation (LLLT vs. control)

Ten studies involving 13 RCTs with 744 participants were included. Compared with the control group, LLLT significantly improved shoulder flexion ROM (MD = 4.35, 95% CI: 1.13 to 7.58, *P* = 0.008) (Fig. 5a), with substantial heterogeneity across the included studies (I² = 95%). Subgroup analysis failed to identify any significant moderating factors for the improvement in shoulder flexion.

(2) Extension

Four studies involving 5 RCTs with 340 participants were included. Relative to the control group, LLLT did not yield a significant effect on shoulder extension ROM (MD = 0.79, 95% CI: −0.47 to 2.06, *P* = 0.22, I² = 74%) (Fig. 5b). Subgroup analysis showed that the combination of LLLT and exercise therapy achieved significant improvements in shoulder extension ROM.

(3) Abduction

Twelve studies involving 15 RCTs with 854 participants were included. Compared with the control group, LLLT significantly improved shoulder abduction ROM (MD = 3.72, 95% CI: 0.79 to 6.64, *P* = 0.01, I² = 95%) (Fig. 5c). Subgroup analysis showed that therapeutic effects at the 3-month follow-up were superior to those at the 1-month follow-up.

(4) External rotation

Eleven studies involving 14 RCTs with 794 participants were included. Compared with the control group, LLLT significantly improved shoulder external rotation ROM (MD = 1.96, 95% CI: 0.51 to 3.42, *P* = 0.008, I² = 83%) (Fig. 5d).

(5) Internal rotation

Seven studies involving 10 RCTs with 576 participants were included. Compared with the control group, LLLT showed no significant effect on shoulder internal rotation ROM (MD = 1.14, 95% CI: −1.51 to 3.78, *P* = 0.40, I² = 95%) (Fig. 5e). Subgroup analysis showed that therapeutic effects at the 1-month follow-up were superior to those at the 3-month follow-up, a temporal trend opposite to that observed for shoulder abduction. Given the lack of overall statistical significance for internal rotation, subgroup results should be interpreted carefully.

#### Meta-regression and subgroup analysis

Regression analysis revealed a stable negative association between the number of treatment sessions and all clinical outcomes. The coefficient for pain was 0.81 (95% CI: 0.15, *P* = 1.46), indicating that more sessions were associated with weaker analgesic effects. The coefficient for SPADI was 0.52, indicating that increased sessions were associated with less improvement in shoulder function. The coefficients for shoulder flexion (β = − 1.08), external rotation (β = − 0.63), and abduction (β = − 0.93) were all negative, indicating that the magnitude of improvement in ROM decreased with more treatment sessions. Subgroup analysis further showed that across all outcomes, effect sizes were higher in the < 12‑session group than in the ≥ 12‑session group, suggesting a possible therapeutic threshold. Although all P values from the analyses were > 0.05, indicating that the number of treatment sessions was not identified as a significant moderator. But this negative association was highly consistent across all outcomes, suggesting a systematic pattern rather than a chance finding due to random error. (see Additional file 6 and Additional file 2).

#### Sensitivity analysis and publication bias

Funnel plots and Egger’s test revealed no significant publication bias (see Additional file 5). Sensitivity analysis revealed that the statistical significance of shoulder abduction disappeared only after excluding the study by Abrisham, indicating that result for this outcome was sensitive to this single study and lacked robustness. After sensitivity analysis, no substantial changes were observed in the pooled effect sizes and CIs for all other outcomes, confirming good robustness of the overall findings.

#### Evidence quality rating

The certainty of evidence for pain, shoulder flexion, abduction and external rotation ROM was graded as low (see Additional file 3). The main reasons for downgrading were substantial heterogeneity across studies (all I² values exceeded 80%) and widespread inadequate blinding, which were regarded as study limitations. The certainty of evidence for shoulder function and disability, as well as shoulder extension and internal rotation ROM, was rated as very low. Beyond the above downgrading factors, the evidence certainty was further downgraded due to the limited number of studies and imprecision indicated by wide CIs around the effect estimates.

## Discussion

This study included 17 studies involving 21 RCTs, representing the largest-scale meta-analysis to date focusing on LLLT for the treatment of SIS. Our findings indicated that LLLT markedly relieved pain and enhanced shoulder flexion, abduction, and external rotation ROM in patients with SIS; however, it did not yield a significant effect on shoulder extension, internal rotation ROM, and overall functional disability. Of note, the pooled effect sizes for all primary outcomes failed to reach the corresponding minimal clinically important difference (MCID) thresholds, and most outcomes showed substantial heterogeneity (I² = 79% to 95%), indicating that the above conclusions are all exploratory findings and should be interpreted with caution upon full recognition of their limitations.

### Comparison with previous studies

Since 2020, only three studies have systematically examined the efficacy of LLLT for SIS, yet all three present notable limitations in research scope and methodology, with their conclusions remaining conflicting. Castaldo et al. only performed a qualitative synthesis and did not conduct quantitative pooling, thus failing to identify the actual efficacy of LLLT [3]. El Tayeb et al. only included 2 RCTs limited to the pairwise comparison between LLLT and ultrasound therapy, and found no significant efficacy differences between the two treatments [10]. However, that study included only two RCTs and therefore lacked sufficient statistical power to detect small-to-moderate effects. Consequently, its null findings should be interpreted with caution rather than taken as definitive evidence. Although Giulia et al. included 10 RCTs, their control interventions were restricted to routine exercise [8]. It should be noted that the three aforementioned reviews share several methodological limitations. These constraints directly reduce the clinical guidance value of their conclusions. First, prior reviews only assessed shoulder ROM in a single plane. Clinically, however, patients with SIS frequently present with motion restrictions in other planes. Second, none of these reviews used appropriate methods to assess the certainty of evidence. Accordingly, clinicians cannot determine the reliability of their conclusions. Third, they failed to report the methods for handling multi-arm trials, which may introduce a risk of statistical bias.

To address the aforementioned limitations, the strengths of our analysis are outlined below: first, prior reviews were limited by small sample sizes and insufficient statistical power, and we included the largest number of RCTs to date (*n* = 17), allowing us to detect clinical differences of small-to-moderate effect sizes; second, while previous reviews only assessed shoulder ROM in a single plane, we systematically evaluated five shoulder motion planes including flexion, extension, abduction, external rotation, and internal rotation, and for the first time, we found that LLLT exerts differential effects across these movements—it improves flexion, abduction and external rotation, but produces no meaningful improvements in extension and internal rotation, a finding that has never been reported in any prior meta-analysis and bears important clinical implications; third, unlike prior reviews that only compared LLLT with exercise therapy, we included all available comparator interventions covering exercise, ultrasound, manual therapy, peloidotherapy and other physical therapy modalities rather than restricting analyses to a single control group, which enables a more comprehensive assessment of the relative efficacy of LLLT across distinct clinical scenarios; fourth, prior reviews did not clearly describe how they managed multi-arm trials, whereas we processed data for multi-arm trials in strict accordance with the standards of the Cochrane Handbook to ensure rigorous and reliable statistical analyses; fifth, unlike previous reviews that omitted the formal evaluation of evidence certainty, we adopted the GRADE system to objectively grade evidence certainty, providing clinicians with clear and transparent standards to assess evidence reliability.

### Pain

This study demonstrated that LLLT significantly reduced VAS pain scores in patients with SIS (MD = − 0.54 cm). Subgroup analysis indicated that LLLT provided similar pain relief for night pain, movement-related pain, and resting pain, and its analgesic effect did not differ based on pain type.

For patients with SIS, pain is the main reason for seeking medical attention. Unbearable pain not only affects mood but also impairs daily activities. The relative analgesic advantage of LLLT provides some evidence for its use as an adjuvant conservative treatment. However, whether its effect size is large enough to bring perceptible clinical improvement for patients remains to be verified by more rigorous study designs. Based on data from 193 patients with SIS, Kanto et al. calculated that the MCID for movement-related VAS pain was 2.0 cm [36]. In comparison, theubacromial pain syndrome pooled effect size of 0.54 cm in this study was far below this threshold, indicating that statistically significant improvement may not be equivalent to clinically significant improvement.

### ROM of the shoulder joint

Our study revealed that LLLT markedly improved shoulder ROM in flexion (MD = 4.35°), abduction (MD = 3.72°), and external rotation (MD = 1.96°), while exerting no significant effect on shoulder extension and internal rotation. Previous studies indicate that shoulder abduction and flexion directly reduce the acromiohumeral distance and significantly elevate contact pressure on subacromial soft tissues, making these motions the most prone to SIS impingement [37]. LLLT can effectively reduce motion resistance in these two directions by alleviating local inflammation and pain, thereby enhancing the corresponding ROM. Biomechanical studies demonstrate that shoulder external rotation moves the greater tubercle of the humerus away from the acromial arch and prevents impingement during shoulder elevation [38]. In patients with SIS, limited external rotation to some extent reflects pain-induced protective muscle inhibition rather than direct mechanical obstruction caused by impingement. The analgesic effect of LLLT can therefore relieve this inhibition and improve external rotation ROM. In addition, limited RCTs on LLLT for shoulder extension and internal rotation ROM lead to insufficient statistical power, so whether LLLT improves these two motion ranges needs further validation.

From a clinical significance perspective, Simovitch et al. reported MCID reference values for ROM in patients undergoing shoulder arthroplasty: approximately 7° for active abduction, 12° for active flexion, and 3° for active external rotation [39]. Against this benchmark, the pooled improvements in flexion (4.35°) and abduction (3.72°) in this study both fell short of the corresponding reference thresholds, and external rotation (1.96°) was also lower than its reference threshold of 3°. However, the aforementioned reference values were derived from surgical populations, who had fundamental differences from patients with SIS receiving conservative treatment. Additionally, no specific investigations on the MCID for ROM in conservatively treated SIS patients are currently available, so the above comparisons only serve as a reference. Overall, these findings suggest that the statistical improvement in joint ROM induced by LLLT does not necessarily equate to the perceived functional benefit for patients.

### Shoulder pain and disability

LLLT showed no significant improvement in SPADI scores (MD = −3.61, *P* = 0.23). The SPADI includes complex daily activities such as grooming, dressing, and weight-bearing. Its improvement relies on the synergistic restoration of multidimensional functions including muscle strength, joint stability, and neuromuscular coordination, rather than isolated modest gains in joint ROM.

Subgroup analyses showed that LLLT combined with exercise therapy or KT improved SPADI scores, whereas no such effect was observed with LLLT alone. Pain relief is the primary mechanism of LLLT. However, this therapy is not able to remedy structural defects or joint instability [40]. For patients with SIS, ultimate functional improvement relies on conventional exercise therapy following pain relief. By correcting abnormal posture and establishing normal movement patterns, comprehensive and durable recovery of shoulder function can be achieved.

### Considerations on the potential inhibitory effects of increased treatment frequency

To further explore the potential threshold effect, we stratified the included studies by the median number of treatment sessions (12 sessions). Although neither meta-regression nor subgroup analysis showed treatment sessions to be a statistically significant factor influencing treatment effects, our study still yielded an unexpected but consistent finding: a negative association between the number of treatment sessions and clinical improvement across multiple outcomes. Subgroup analysis further showed that, across all outcomes, the effect sizes of studies with fewer than 12 sessions were consistently larger than those of studies with 12 or more sessions. Although the intuitive assumption in clinical practice is that “more is better,” our results are consistent with a growing body of evidence suggesting that the dose–response relationship in physical therapy and rehabilitation is considerably more complex than a simple linear model.

First, the biphasic dose-response theory may explain this phenomenon: low to moderate frequencies and intensities of physical therapy produce therapeutic effects, whereas excessive sessions and doses may inhibit recovery and even cause harm [41]. This pattern has been validated in LLLT and exercise rehabilitation. Moderate therapies enhance tissue repair, while overtherapies can overwhelm antioxidant defenses and cause tissue damage [42, 43].

Second, the therapeutic window theory posits that interventions are most effective within a specific range of session numbers; too few or too many sessions reduce efficacy and may aggravate symptoms [44]. In rehabilitation, this effective window lies between insufficient stimulation and overtraining, with excessive treatment leading to fatigue, injury, and maladaptation. Based on our data, we speculate that some patients in our cohort may have exceeded the upper limit of this effective window, resulting in diminished or even negative returns. This interpretation is supported by evidence that for most musculoskeletal conditions, long-term improvement does not continue to increase with more sessions [45].

Third, from a biopsychosocial perspective, frequent treatment visits may foster patient dependency and reduce active self-exercise, which can prolong recovery, whereas self-management is key to long-term rehabilitation [46].

It should be noted that our study can only demonstrate an association, not causality. This negative correlation could also be due to confounding by indication. Patients with more severe or complex conditions may have received more sessions, yet their recovery remained poor despite additional treatment. Nevertheless, the consistent negative trend across all observed outcomes provides important information and can guide future research on optimal treatment frequency. Further prospective studies are needed to identify the threshold beyond which additional sessions cease to be beneficial or become counterproductive [47].

### Limitations

First, significant heterogeneity was observed across all three outcomes, compromising the reliability of results. We speculate that the heterogeneity may stem from multiple combined factors. Firstly, there are fundamental differences in intervention measures, as laser therapy, exercise therapy, and ultrasound therapy target entirely distinct clinical comparative questions. Meanwhile, LLLT parameters varied greatly across the included trials, and different parameters may produce completely different biological effects. However, the limited number of included studies prevented us from conducting further subgroup analyses on these parameters. Furthermore, only a few studies reported whether joint ROM was measured actively or passively, while most studies did not specify this information. We could not separately clarify the effects of LLLT on active and passive shoulder ROM, which is likely one potential source of high heterogeneity for ROM related outcomes.

Second, the included studies showed substantial differences in blinding implementation, and potential publication bias may exist.

Third, each subgroup contained too few studies, resulting in insufficient statistical power. Therefore, the results should be regarded as exploratory findings.

Fourth, no adjustments were made for multiple testing across outcomes and covariates, which may increase the risk of type I error; therefore, our findings should be interpreted as exploratory rather than confirmatory.

### Future research directions

First, more future studies should focus on the optimization and unified standardization of LLLT parameters, including wavelength, output power and frequency. Second, subgroup analyses show that LLLT combined with exercise therapy yields significant improvements in shoulder disability. However, limited by the small sample size of subgroups, the relevant conclusions remain unrobust. Future researchers should conduct RCTs with adequate sample sizes and sufficient follow-up duration for different combined regimens to clarify the optimal intervention strategy. Third, Hernán Andrés et al. reported that HILT reduced the VAS score by an average of 1.4 cm in patients with SIS, with a markedly larger effect size than that in the present study. Although HILT produces prominent therapeutic effects, it is a relatively novel therapy and differs greatly from LLLT in parameter settings and mechanisms of action. At present, there are few studies comparing the efficacy of LLLT and HILT, which deserves focused attention in future research. Fourth, future studies are required to differentiate active and passive shoulder ROM and implement standardized procedures during measurement, so as to reduce inter-study heterogeneity. Fifth, there are currently no specialized studies on the MCID of ROM among SIS patients receiving conservative treatment. Establishing corresponding threshold values is essential for accurately evaluating the clinical significance of conservative treatment and represents an important research gap in future clinimetric studies. Sixth, future studies should explore the dose-response relationship between treatment sessions and clinical outcomes to determine the optimal therapeutic window. Our findings suggest that 12 sessions may represent the upper limit of the effective range; however, this threshold requires prospective validation to better inform the clinical application of LLLT.

## Conclusions

Low-quality evidence indicates that LLLT can effectively relieve pain symptoms in patients with SIS and markedly improve shoulder flexion, abduction, and external rotation ROM. Very low-quality evidence suggests that LLLT has no significant beneficial effect on shoulder functional disability and also shows no notable therapeutic effect on shoulder extension and internal rotation ROM. However, the pooled effect sizes for most positive outcomes did not reach the corresponding MCID thresholds. This indicates that statistical significance does not equate to patient-perceptible clinical benefit, and clinical decisions should be made with caution based on individual patient conditions. Given the notable methodological and clinical heterogeneity among the studies, the conclusions should only serve as an exploratory reference. In addition, based on our findings, we suggest that more than about 12 sessions may not add further benefit and could even be harmful. Clinicians may reassess whether to continue therapy when sessions approach this number, rather than always extending treatment. Of note, this suggestion is exploratory and based on indirect evidence, not a firm recommendation. Future studies should confirm the optimal session number and examine why individual responses may differ.

## Supplementary Information

Below is the link to the electronic supplementary material.

Supplementary Material 1 (DOCX 16.7 KB)

Supplementary Material 2 (XLSX 14.0 KB)

Supplementary Material 3 (DOCX 21.3 KB)

Supplementary Material 4 (PDF 99.0 KB)

Supplementary Material 5 (DOCX 1.45 MB)

Supplementary Material 6 (XLSX 11.1 KB)

## Data Availability

All data are available.

## References

[1] Hodgetts C, Walker B (2021) Epidemiology, common diagnoses, treatments and prognosis of shoulder pain: a narrative review. Int J Osteopath Med 42:11–9. 10.1016/j.ijosm.2021.10.006

[2] Greving K, Dorrestijn O, Winters JC, Groenhof F, van der Meer K, Stevens M et al (2012) Incidence, prevalence, and consultation rates of shoulder complaints in general practice. Scand J Rheumatol 41(2):150–155. 10.3109/03009742.2011.605390 21936616

[3] Castaldo M, De Angelis D’Ossat A, Gnessi P, Galeoto G (2023) A systematic review on low-level laser therapy in the management of shoulder impingement syndrome. Appl Sci 13(6):3536. 10.3390/app13063536

[4] Hanchard NC, Lenza M, Handoll HH, Takwoingi Y (2013) Physical tests for shoulder impingements and local lesions of bursa, tendon or labrum that may accompany impingement. Cochrane Database Syst Rev 2013(4):Cd007427. 10.1002/14651858.CD007427.pub2 23633343 PMC6464770

[5] Chaimongkhol T, Benjachaya S, Mahakkanukrauh P (2020) Acromial morphology and morphometry associated with subacromial impingement syndrome. Anat Cell Biol 53(4):435–443. 10.5115/acb.20.166 32963132 PMC7769113

[6] Garving C, Jakob S, Bauer I, Nadjar R, Brunner UH (2017) Impingement Syndrome of the Shoulder. Deutsches Arzteblatt Int 114(45):765–776. 10.3238/arztebl.2017.0765 PMC5729225 29202926

[7] Consigliere P, Haddo O, Levy O, Sforza G (2018) Subacromial impingement syndrome: management challenges. Orthop Res Rev 10:83–91 30774463 10.2147/ORR.S157864 PMC6376459

[8] de Lara Quagliotto G, Manchope MP, Hilario R, Zubeldia V, Stacheslki RA, de Carvalho AR et al (2026) Photobiomodulation associated with physical exercise in shoulder impingement syndrome. Systematic review with meta-analysis. Photochem Photobiol 102(1):220–236. 10.1111/php.14113 40365684 PMC12807334

[9] Clijsen R, Brunner A, Barbero M, Clarys P, Taeymans J (2017) Effects of low-level laser therapy on pain in patients with musculoskeletal disorders: a systematic review and meta-analysis. Eur J Phys Rehabil Med 53(4):603–610. 10.23736/s1973-9087.17.04432-x 28145397

[10] El Tayeb MN, Hassan AA, Abuelnaga YG, Eid PM, Tarkhan YM, Fathallah MM et al (2021) Low level laser therapy versus ultrasonic therapy for the treatment of shoulder impingement syndrome a systematic review of randomized controlled trials. QJM. 10.1093/qjmed/hcab116

[11] Tashjian RZ, Deloach J, Porucznik CA, Powell AP (2009) Minimal clinically important differences (MCID) and patient acceptable symptomatic state (PASS) for visual analog scales (VAS) measuring pain in patients treated for rotator cuff disease. J Shoulder Elb Surg 18(6):927–932. 10.1016/j.jse.2009.03.021 19535272

[12] Breckenridge JD, McAuley JH (2011) Shoulder Pain and Disability Index (SPADI). J Physiother 57(3):197. 10.1016/s1836-9553(11)70045-5 21843839

[13] Pérez-de la Cruz S, de León ÓA, Mallada NP, Rodríguez AV (2021) Validity and intra-examiner reliability of the Hawk goniometer versus the universal goniometer for the measurement of range of motion of the glenohumeral joint. Med Eng Phys 89:7–11. 10.1016/j.medengphy.2021.01.005 33608127

[14] Wan X, Wang W, Liu J, Tong T (2014) Estimating the sample mean and standard deviation from the sample size, median, range and/or interquartile range. BMC Med Res Methodol 14:135. 10.1186/1471-2288-14-135 25524443 PMC4383202

[15] Yan L, Li D, Xing D, Fan Z, Du G, Jiu J et al (2025) Comparative efficacy and safety of exercise modalities in knee osteoarthritis: systematic review and network meta-analysis. BMJ 391:e085242. 10.1136/bmj-2025-085242IF:55.1Q1B1. https://europepmc.org/articles/PMC4383202IF: 3.7 Q2 B2?pdf=render 41093618 PMC12522397

[16] Alfredo PP, Bjordal JM, Junior WS, Marques AP, Casarotto RA (2021) Efficacy of low-level laser therapy combined with exercise for subacromial impingement syndrome: A randomised controlled trial. Clin Rehabil 35(6):851–860. 10.1177/0269215520980984 33307783

[17] Calis HT, Berberoglu N, Calis M (2011) Are ultrasound, laser and exercise superior to each other in the treatment of subacromial impingement syndrome? A randomized clinical trial. Eur J Phys Rehabil Med 47(3):375–380 21946399

[18] Ekin Ilke Sen SA, Narangerel Tseveendorj Elçim, Yılmaz A, Oral N Capan (2023) Low-level laser therapy versus ultrasound therapy combined with home-based exercise in patients with subacromial impingement syndrome: A randomized-controlled trial. Turk J Phys Med Rehab 424–433 10.5606/tftrd.2023.11193 PMC11099852 38766575

[19] Agir FC, Karpuz S, Yilmaz R, Akkurt HE, Yilmaz H (2024) Comparison of the efficacy of low intensity laser and peloid therapy in patients with subacromial impingement syndrome. Int J Biometeorol 68(8):1507–1517. 10.1007/s00484-024-02660-2 38953979

[20] DerSimonian R, Laird N (2015) Meta-analysis in clinical trials revisited. Contemp Clin Trials 45(Pt A):139–45. 10.1016/j.cct.2015.09.002 26343745 PMC4639420

[21] Egger M, Davey Smith G, Schneider M, Minder C (1997) Bias in meta-analysis detected by a simple, graphical test. BMJ 315(7109):629–634. 10.1136/bmj.315.7109.629 9310563 PMC2127453

[22] Higgins JP, Altman DG, Gøtzsche PC, Jüni P, Moher D, Oxman AD et al (2011) The Cochrane Collaboration’s tool for assessing risk of bias in randomised trials. BMJ 343:d5928. 10.1136/bmj.d5928 22008217 PMC3196245

[23] Balshem H, Helfand M, Schünemann HJ, Oxman AD, Kunz R, Brozek J et al (2011) GRADE guidelines: 3. Rating the quality of evidence. J Clin Epidemiol 64(4):401–406. 10.1016/j.jclinepi.2010.07.015 21208779

[24] Bal A, Eksioglu E, Gurcay E, Gulec B, Karaahmet O, Cakci A (2009) Low-level laser therapy in subacromial impingement syndrome. Photomed Laser Surg 27(1):31–36. 10.1089/pho.2007.2222 19250050

[25] Yeldan I, Cetin E, Ozdincler AR (2009) The effectiveness of low-level laser therapy on shoulder function in subacromial impingement syndrome. Disabil Rehabil 31(11):935–940. 10.1080/09638280802377985 19031167

[26] Dogan SK, Ay S, Evcik D (2010) The effectiveness of low laser therapy in subacromial impingement syndrome: a randomized placebo controlled double-blind prospective study. Clin (Sao Paulo) 65(10):1019–1022. 10.1590/s1807-59322010001000016 PMC2972614 21120304

[27] Abrisham SM, Kermani-Alghoraishi M, Ghahramani R, Jabbari L, Jomeh H, Zare M (2011) Additive effects of low-level laser therapy with exercise on subacromial syndrome: a randomised, double-blind, controlled trial. Clin Rheumatol 30(10):1341–1346. 10.1007/s10067-011-1757-7 21538218

[28] Mariam Abd El-Rhman Mohamed, Abd-Allah HME-A, Maha Mostafa M (2017) Low level laser therapy versus eccentric exercises in the treatment of shoulder impingement syndrome. J Adv Pharm Edu Res 428–437

[29] Aygül Özmen SA (2015) Comparing of the Efficacy and Effectiveness of Isokinetic Exercise, Laser, Pseudoiontophoresis and Iontophoresis Treatments According to the Stage in Treatments of Subacromial Impingement Syndrome. Turkiye Klinikleri J Med Sci 166–178

[30] Pınar Atıcı Öztürk İŞ Altınay Göksel Karatepe, Taciser Kaya, Rezzan Günaydın (2015) Effectiveness of low-level laser therapy in patients with subacromial impingement syndrome: A randomized, placebo controlled, prospective study. Tepecik Eğitim ve Araştırma Hastanesi Dergisi. 78–84

[31] Ravish VNRHD, Sharath U, R D, Deva Paul, Doss (2015) To Compare the Effectiveness of Therapeutic Kinesio Taping and Exercises and Ultrasound with Therapeutic Kinesio Taping and Exercises and Low Intensity Laser in Treating Patients with Subacromial Impingement Syndrome. J Evol Med Dent Sci 6303–6314

[32] Zohreh Zaki RR, Schmitz M, Kambiz Abbasi (2021) Comparison of low level and high power laser combined with kinesiology taping on shoulder function and musculoskeletal sonography parameters in subacromial impingement syndrome: a Randomized placebo-controlled trial. Physiotherapy Theory and Practice 10.1080/09593985.2021.1934926 34184965

[33] Saleh MS, Galal DOS, Ali MS, Ibrahim DI (2025) High-intensity versus low-level laser therapy in treatment of patients with subacromial impingement syndrome: a randomized, double-blind, controlled trial. Lasers Med Sci 40(1):8. 10.1007/s10103-024-04262-1 39757334

[34] Badıl Güloğlu S (2021) Comparison of low-level laser treatment and extracorporeal shock wave therapy in subacromial impingement syndrome: a randomized, prospective clinical study. Lasers Med Sci 36(4):773–781. 10.1007/s10103-020-03093-0 32638239

[35] Eslamian F, Shakouri SK, Ghojazadeh M, Nobari OE, Eftekharsadat B (2012) Effects of low-level laser therapy in combination with physiotherapy in the management of rotator cuff tendinitis. Lasers Med Sci 27(5):951–958. 10.1007/s10103-011-1001-3 22052627

[36] Somashekhar RR, Brahma KK, Ranganath HD, Yatish R, Vivek J (2014) To compare the efficacy of ultrasound therapy with exercises versus laser therapy with exercises in the treatment of subacromial impingement syndrome. J Evol Med Dent Sci 10314–10324

[37] Kanto K, Lähdeoja T, Paavola M, Aronen P, Järvinen TLN, Jokihaara J et al (2021) Minimal important difference and patient acceptable symptom state for pain, Constant-Murley score and Simple Shoulder Test in patients with subacromial pain syndrome. BMC Med Res Methodol 21(1):45. 10.1186/s12874-021-01241-w 33676417 PMC7937213

[38] Yanai T, Fuss FK, Fukunaga T (2006) In vivo measurements of subacromial impingement: Substantial compression develops in abduction with large internal rotation. Clin Biomech Elsevier Ltd 21(7):692–700. 10.1016/j.clinbiomech.2006.03.001 16632128

[39] Reinold MM, Escamilla RF, Wilk KE (2009) Current concepts in the scientific and clinical rationale behind exercises for glenohumeral and scapulothoracic musculature. J Orthop Sports Phys Ther 39(2):105–117. 10.2519/jospt.2009.2835 19194023

[40] Simovitch R, Flurin PH, Wright T, Zuckerman JD, Roche CP (2018) Quantifying success after total shoulder arthroplasty: the minimal clinically important difference. J Shoulder Elb Surg 27(2):298–305. 10.1016/j.jse.2017.09.013 29162305

[41] Cotler HB, Chow RT, Hamblin MR, Carroll J (2015) The Use of Low Level Laser Therapy (LLLT) For Musculoskeletal Pain. MOJ Orthop Rheumatol 2(5):00068. 10.15406/mojor.2015.02.00068 26858986 PMC4743666

[42] Huang YY, Chen AC, Carroll JD, Hamblin MR (2009) Biphasic dose response in low level light therapy. Dose Response 7(4):358–383. 10.2203/dose-response.09-027.Hamblin 20011653 PMC2790317

[43] Demidova-Rice TN, Salomatina EV, Yaroslavsky AN, Herman IM, Hamblin MR (2007) Low-level light stimulates excisional wound healing in mice. Lasers Surg Med 39(9):706–715. 10.1002/lsm.20549 17960752 PMC2935798

[44] Radak Z, Ishihara K, Tekus E, Varga C, Posa A, Balogh L et al (2017) Exercise, oxidants, and antioxidants change the shape of the bell-shaped hormesis curve. Redox Biol 12:285–290. 10.1016/j.redox.2017.02.015 28285189 PMC5345970

[45] Foster C, Cortis C, Fusco A (2021) Exercise evaluation and prescription. J Funct Morphol Kinesiol. 10.3390/jfmk6010031 33806860 PMC8006239

[46] Murtagh S, Touloumis A, Olivier G, Butterworth J, Hammerbeck U (2024) Physiotherapy Outcomes Are Associated With Shorter Waiting Times, More Treatment Sessions and Younger Age: Analysis of a Clinical Database. Musculoskelet Care 22(3):e1924. 10.1002/msc.1924 39134408

[47] Hutting N (2025) From passive care to self-management support: A new era in manual and musculoskeletal physiotherapy. Musculoskelet Sci Pract 78:103370. 10.1016/j.msksp.2025.103370 40543106

