# Supplementary material for: Efficacy of low-level laser therapy for subacromial impingement syndrome: a meta-analysis of randomized controlled trials
Source: Lasers Med Sci. 2026 Sep 14;41(1):228. doi: 10.1007/s10103-026-05028-7 (PMC13572333; doi:10.1007/s10103-026-05028-7)
Supplement: Supplementary file 2 [file 10103_2026_5028_MOESM1_ESM.docx]

Pubmed(36)

#1 Search: **"Shoulder Impingement Syndrome"[Mesh]** Sort by: **Most Recent	2108**

**#2** Search: **"Shoulder Impingement Syndromes"[Title/Abstract] OR "Rotator Cuff Impingement Syndrome"[Title/Abstract] OR "Rotator Cuff Impingement"[Title/Abstract] OR "Impingement, Rotator Cuff"[Title/Abstract] OR "Impingements, Rotator Cuff"[Title/Abstract] OR "Rotator Cuff Impingements"[Title/Abstract] OR "Shoulder Impingement"[Title/Abstract] OR "Impingement, Shoulder"[Title/Abstract] OR "Shoulder Impingements"[Title/Abstract] OR "Coracohumeral Impingement Syndrome"[Title/Abstract] OR "Coracohumeral Impingement Syndromes"[Title/Abstract] OR "Subacromial Impingement Syndrome"[Title/Abstract] OR "Subacromial Impingement Syndromes"[Title/Abstract] OR "Coracohumeral Impingement"[Title/Abstract] OR "Coracohumeral Impingements"[Title/Abstract] OR "Impingement, Coracohumeral"[Title/Abstract] OR "Coracoid Impingement Syndrome"[Title/Abstract] OR "Coracoid Impingement Syndromes"[Title/Abstract] OR "Outlet Impingement Syndrome"[Title/Abstract] OR "Outlet Impingement Syndromes"[Title/Abstract] OR "Outlet Impingement"[Title/Abstract] OR "Outlet Impingements"[Title/Abstract] OR "Internal Impingement Syndrome"[Title/Abstract] OR "Internal Impingement Syndromes"[Title/Abstract] OR "Posterosuperior Glenoid Impingement"[Title/Abstract] OR "Impingement, Posterosuperior Glenoid"[Title/Abstract] OR "Impingements, Posterosuperior Glenoid"[Title/Abstract] OR "Posterosuperior Glenoid Impingements"[Title/Abstract]** Sort by: **Most Recent 1527**

#3 Search: **("Shoulder Impingement Syndrome"[Mesh]) OR ("Shoulder Impingement Syndromes"[Title/Abstract] OR "Rotator Cuff Impingement Syndrome"[Title/Abstract] OR "Rotator Cuff Impingement"[Title/Abstract] OR "Impingement, Rotator Cuff"[Title/Abstract] OR "Impingements, Rotator Cuff"[Title/Abstract] OR "Rotator Cuff Impingements"[Title/Abstract] OR "Shoulder Impingement"[Title/Abstract] OR "Impingement, Shoulder"[Title/Abstract] OR "Shoulder Impingements"[Title/Abstract] OR "Coracohumeral Impingement Syndrome"[Title/Abstract] OR "Coracohumeral Impingement Syndromes"[Title/Abstract] OR "Subacromial Impingement Syndrome"[Title/Abstract] OR "Subacromial Impingement Syndromes"[Title/Abstract] OR "Coracohumeral Impingement"[Title/Abstract] OR "Coracohumeral Impingements"[Title/Abstract] OR "Impingement, Coracohumeral"[Title/Abstract] OR "Coracoid Impingement Syndrome"[Title/Abstract] OR "Coracoid Impingement Syndromes"[Title/Abstract] OR "Outlet Impingement Syndrome"[Title/Abstract] OR "Outlet Impingement Syndromes"[Title/Abstract] OR "Outlet Impingement"[Title/Abstract] OR "Outlet Impingements"[Title/Abstract] OR "Internal Impingement Syndrome"[Title/Abstract] OR "Internal Impingement Syndromes"[Title/Abstract] OR "Posterosuperior Glenoid Impingement"[Title/Abstract] OR "Impingement, Posterosuperior Glenoid"[Title/Abstract] OR "Impingements, Posterosuperior Glenoid"[Title/Abstract] OR "Posterosuperior Glenoid Impingements"[Title/Abstract])** Sort by: **Most Recent 2779**

**#4** Search: **"Laser Therapy"[Mesh]** Sort by: **Most Recent 71832**

**#5** Search: **"Laser Therapies"[Title/Abstract] OR "Therapies, Laser"[Title/Abstract] OR "Therapy, Laser"[Title/Abstract] OR "Laser Scalpel"[Title/Abstract] OR "Laser Scalpels"[Title/Abstract] OR "Scalpel, Laser"[Title/Abstract] OR "Scalpels, Laser"[Title/Abstract] OR "Laser Knives"[Title/Abstract] OR "Knive, Laser"[Title/Abstract] OR "Knives, Laser"[Title/Abstract] OR "Laser Knive"[Title/Abstract] OR "Laser Knife"[Title/Abstract] OR "Knife, Laser"[Title/Abstract] OR "Knifes, Laser"[Title/Abstract] OR "Laser Knifes"[Title/Abstract] OR "Laser Surgery"[Title/Abstract] OR "Laser Surgeries"[Title/Abstract] OR "Surgeries, Laser"[Title/Abstract] OR "Surgery, Laser"[Title/Abstract] OR "Vaporization, Laser"[Title/Abstract] OR "Laser Vaporization"[Title/Abstract] OR "Pulsed Laser Tissue Ablation"[Title/Abstract] OR "Laser Photoablation of Tissue"[Title/Abstract] OR "Laser Tissue Ablation"[Title/Abstract] OR "Ablation, Laser Tissue"[Title/Abstract] OR "Tissue Ablation, Laser"[Title/Abstract] OR "Laser Ablation"[Title/Abstract] OR "Ablation, Laser"[Title/Abstract] OR "Nonablative Laser Treatment"[Title/Abstract] OR "Laser Treatment, Nonablative"[Title/Abstract] OR "Laser Treatments, Nonablative"[Title/Abstract] OR "Nonablative Laser Treatments"[Title/Abstract]** Sort by: **Most Recent 17824**

**#6** Search: **("Laser Therapy"[Mesh]) OR ("Laser Therapies"[Title/Abstract] OR "Therapies, Laser"[Title/Abstract] OR "Therapy, Laser"[Title/Abstract] OR "Laser Scalpel"[Title/Abstract] OR "Laser Scalpels"[Title/Abstract] OR "Scalpel, Laser"[Title/Abstract] OR "Scalpels, Laser"[Title/Abstract] OR "Laser Knives"[Title/Abstract] OR "Knive, Laser"[Title/Abstract] OR "Knives, Laser"[Title/Abstract] OR "Laser Knive"[Title/Abstract] OR "Laser Knife"[Title/Abstract] OR "Knife, Laser"[Title/Abstract] OR "Knifes, Laser"[Title/Abstract] OR "Laser Knifes"[Title/Abstract] OR "Laser Surgery"[Title/Abstract] OR "Laser Surgeries"[Title/Abstract] OR "Surgeries, Laser"[Title/Abstract] OR "Surgery, Laser"[Title/Abstract] OR "Vaporization, Laser"[Title/Abstract] OR "Laser Vaporization"[Title/Abstract] OR "Pulsed Laser Tissue Ablation"[Title/Abstract] OR "Laser Photoablation of Tissue"[Title/Abstract] OR "Laser Tissue Ablation"[Title/Abstract] OR "Ablation, Laser Tissue"[Title/Abstract] OR "Tissue Ablation, Laser"[Title/Abstract] OR "Laser Ablation"[Title/Abstract] OR "Ablation, Laser"[Title/Abstract] OR "Nonablative Laser Treatment"[Title/Abstract] OR "Laser Treatment, Nonablative"[Title/Abstract] OR "Laser Treatments, Nonablative"[Title/Abstract] OR "Nonablative Laser Treatments"[Title/Abstract])** Sort by: **Most Recent 82024**

**#7** Search: **(("Laser Therapy"[Mesh]) OR ("Laser Therapies"[Title/Abstract] OR "Therapies, Laser"[Title/Abstract] OR "Therapy, Laser"[Title/Abstract] OR "Laser Scalpel"[Title/Abstract] OR "Laser Scalpels"[Title/Abstract] OR "Scalpel, Laser"[Title/Abstract] OR "Scalpels, Laser"[Title/Abstract] OR "Laser Knives"[Title/Abstract] OR "Knive, Laser"[Title/Abstract] OR "Knives, Laser"[Title/Abstract] OR "Laser Knive"[Title/Abstract] OR "Laser Knife"[Title/Abstract] OR "Knife, Laser"[Title/Abstract] OR "Knifes, Laser"[Title/Abstract] OR "Laser Knifes"[Title/Abstract] OR "Laser Surgery"[Title/Abstract] OR "Laser Surgeries"[Title/Abstract] OR "Surgeries, Laser"[Title/Abstract] OR "Surgery, Laser"[Title/Abstract] OR "Vaporization, Laser"[Title/Abstract] OR "Laser Vaporization"[Title/Abstract] OR "Pulsed Laser Tissue Ablation"[Title/Abstract] OR "Laser Photoablation of Tissue"[Title/Abstract] OR "Laser Tissue Ablation"[Title/Abstract] OR "Ablation, Laser Tissue"[Title/Abstract] OR "Tissue Ablation, Laser"[Title/Abstract] OR "Laser Ablation"[Title/Abstract] OR "Ablation, Laser"[Title/Abstract] OR "Nonablative Laser Treatment"[Title/Abstract] OR "Laser Treatment, Nonablative"[Title/Abstract] OR "Laser Treatments, Nonablative"[Title/Abstract] OR "Nonablative Laser Treatments"[Title/Abstract])) AND (("Shoulder Impingement Syndrome"[Mesh]) OR ("Shoulder Impingement Syndromes"[Title/Abstract] OR "Rotator Cuff Impingement Syndrome"[Title/Abstract] OR "Rotator Cuff Impingement"[Title/Abstract] OR "Impingement, Rotator Cuff"[Title/Abstract] OR "Impingements, Rotator Cuff"[Title/Abstract] OR "Rotator Cuff Impingements"[Title/Abstract] OR "Shoulder Impingement"[Title/Abstract] OR "Impingement, Shoulder"[Title/Abstract] OR "Shoulder Impingements"[Title/Abstract] OR "Coracohumeral Impingement Syndrome"[Title/Abstract] OR "Coracohumeral Impingement Syndromes"[Title/Abstract] OR "Subacromial Impingement Syndrome"[Title/Abstract] OR "Subacromial Impingement Syndromes"[Title/Abstract] OR "Coracohumeral Impingement"[Title/Abstract] OR "Coracohumeral Impingements"[Title/Abstract] OR "Impingement, Coracohumeral"[Title/Abstract] OR "Coracoid Impingement Syndrome"[Title/Abstract] OR "Coracoid Impingement Syndromes"[Title/Abstract] OR "Outlet Impingement Syndrome"[Title/Abstract] OR "Outlet Impingement Syndromes"[Title/Abstract] OR "Outlet Impingement"[Title/Abstract] OR "Outlet Impingements"[Title/Abstract] OR "Internal Impingement Syndrome"[Title/Abstract] OR "Internal Impingement Syndromes"[Title/Abstract] OR "Posterosuperior Glenoid Impingement"[Title/Abstract] OR "Impingement, Posterosuperior Glenoid"[Title/Abstract] OR "Impingements, Posterosuperior Glenoid"[Title/Abstract] OR "Posterosuperior Glenoid Impingements"[Title/Abstract]))** Sort by: **Most Recent 36**

Embase(62)

#1 ('shoulder impingement syndrome'/exp OR 'shoulder impingement syndrome') AND [embase]/lim

[3,357](https://embase.publinks.top/)

#2 'rotator cuff impingement':ti,ab,kw OR 'rotator cuff syndrome':ti,ab,kw OR 'rotator cuff tendinitis':ti,ab,kw OR 'rotator cuff tendonitis':ti,ab,kw OR 'shoulder cuff syndrome':ti,ab,kw OR 'shoulder impingement':ti,ab,kw OR 'subacromial impingement':ti,ab,kw OR 'subacromial impingement syndrome':ti,ab,kw OR 'syndrome, shoulder impingement':ti,ab,kw OR 'shoulder impingement syndrome':ti,ab,kw

[3,182](https://embase.publinks.top/)

#3 #1 OR #2

[4,848](https://embase.publinks.top/)

#4 ('laser therapy'/exp OR 'laser therapy') AND [embase]/lim

[36,808](https://embase.publinks.top/)

#5 (((laser:ti,ab,kw AND 'light amplification by stimulated emission of radiation':ti,ab,kw AND therapy:ti,ab,kw OR 'laser treatment':ti,ab,kw OR 'light amplification by stimulated emission of radiation':ti,ab,kw) AND laser:ti,ab,kw AND therapy:ti,ab,kw OR 'light amplification by stimulated emission of radiation therapy':ti,ab,kw) AND 'laser therapy':ti,ab,kw OR 'laser therapy':ti,ab,kw) AND [embase]/lim

[14,493](https://embase.publinks.top/)

#6 #4 OR #5

[36,808](https://embase.publinks.top/)

#7 #3 AND #6

[62](https://embase.publinks.top/)

Cochrane Library(47)

#1 MeSH descriptor: [Shoulder Impingement Syndrome] explode all trees	583

#2 (Coracohumeral Impingement Syndromes or Coracohumeral Impingement Syndrome or Subacromial Impingement Syndromes or Subacromial Impingement Syndrome or Impingement, Coracohumeral or Coracohumeral Impingement or Coracoid Impingement Syndrome or Coracohumeral Impingements or Coracoid Impingement Syndromes or Outlet Impingement Syndrome or Outlet Impingement or Outlet Impingements or Outlet Impingement Syndromes or Internal Impingement Syndromes or Posterosuperior Glenoid Impingements or Internal Impingement Syndrome or Impingements, Posterosuperior Glenoid or Impingement, Posterosuperior Glenoid or Posterosuperior Glenoid Impingement or Shoulder Impingement or Impingements, Rotator Cuff or Shoulder Impingements or Impingement, Shoulder or Impingement, Rotator Cuff or Rotator Cuff Impingements or Rotator Cuff Impingement or Shoulder Impingement Syndromes or Rotator Cuff Impingement Syndrome):ti,ab,kw 1349

#3 #1 or #2 1349

#4 MeSH descriptor: [Laser Therapy] explode all trees 6283

#5 (Therapies, Laser or Laser Therapies or Therapy, Laser or Surgeries, Laser or Surgery, Laser or Laser Surgery or Laser Surgeries or Laser Knives or Laser Knifes or Scalpels, Laser or Knifes, Laser or Knives, Laser or Knife, Laser or Laser Knife or Laser Scalpels or Laser Knive or Scalpel, Laser or Knive, Laser or Laser Scalpel or Laser Photoablation of Tissue or Laser Vaporization or Ablation, Laser Tissue or Laser Ablation or Vaporization, Laser or Pulsed Laser Tissue Ablation or Laser Tissue Ablation or Ablation, Laser or Tissue Ablation, Laser or Laser Treatment, Nonablative or Laser Treatments, Nonablative or Nonablative Laser Treatments or Nonablative Laser Treatment):ti,ab,kw 20311

#6 #4 or #5 20603

#3 and #6 47

Web of science(55)

**#1 (TI=(Shoulder Impingement Syndrome or Shoulder Impingement Syndromes or Rotator Cuff Impingement Syndrome or Rotator Cuff Impingement or Impingement, Rotator Cuff or Impingements, Rotator Cuff or Rotator Cuff Impingements or Shoulder Impingement or Impingement, Shoulder or Shoulder Impingements or Coracohumeral Impingement Syndrome or Coracohumeral Impingement Syndromes or Subacromial Impingement Syndrome or Subacromial Impingement Syndromes or Coracohumeral Impingement or Coracohumeral Impingements or Impingement, Coracohumeral or Coracoid Impingement Syndrome or Coracoid Impingement Syndromes or Outlet Impingement Syndrome or Outlet Impingement Syndromes or Outlet Impingement or Outlet Impingements or Internal Impingement Syndrome or Internal Impingement Syndromes or Posterosuperior Glenoid Impingement or Impingement, Posterosuperior Glenoid or Impingements, Posterosuperior Glenoid or Posterosuperior Glenoid Impingements)) OR AB=(Shoulder Impingement Syndrome or Shoulder Impingement Syndromes or Rotator Cuff Impingement Syndrome or Rotator Cuff Impingement or Impingement, Rotator Cuff or Impingements, Rotator Cuff or Rotator Cuff Impingements or Shoulder Impingement or Impingement, Shoulder or Shoulder Impingements or Coracohumeral Impingement Syndrome or Coracohumeral Impingement Syndromes or Subacromial Impingement Syndrome or Subacromial Impingement Syndromes or Coracohumeral Impingement or Coracohumeral Impingements or Impingement, Coracohumeral or Coracoid Impingement Syndrome or Coracoid Impingement Syndromes or Outlet Impingement Syndrome or Outlet Impingement Syndromes or Outlet Impingement or Outlet Impingements or Internal Impingement Syndrome or Internal Impingement Syndromes or Posterosuperior Glenoid Impingement or Impingement, Posterosuperior Glenoid or Impingements, Posterosuperior Glenoid or Posterosuperior Glenoid Impingements) 3669**

**#2 (TI=(Laser Therapy or Laser Therapies or Therapies, Laser or Therapy, Laser or Laser Scalpel or Laser Scalpels or Scalpel, Laser or Scalpels, Laser or Laser Knives or Knive, Laser or Knives, Laser or Laser Knive or Laser Knife or Knife, Laser or Knifes, Laser or Laser Knifes or Laser Surgery or Laser Surgeries or Surgeries, Laser or Surgery, Laser or Vaporization, Laser or Laser Vaporization or Pulsed Laser Tissue Ablation or Laser Photoablation of Tissue or Laser Tissue Ablation or Ablation, Laser Tissue or Tissue Ablation, Laser or Laser Ablation or Ablation, Laser or Nonablative Laser Treatment or Laser Treatment, Nonablative or Laser Treatments, Nonablative or Nonablative Laser Treatments)) OR AB=(Laser Therapy or Laser Therapies or Therapies, Laser or Therapy, Laser or Laser Scalpel or Laser Scalpels or Scalpel, Laser or Scalpels, Laser or Laser Knives or Knive, Laser or Knives, Laser or Laser Knive or Laser Knife or Knife, Laser or Knifes, Laser or Laser Knifes or Laser Surgery or Laser Surgeries or Surgeries, Laser or Surgery, Laser or Vaporization, Laser or Laser Vaporization or Pulsed Laser Tissue Ablation or Laser Photoablation of Tissue or Laser Tissue Ablation or Ablation, Laser Tissue or Tissue Ablation, Laser or Laser Ablation or Ablation, Laser or Nonablative Laser Treatment or Laser Treatment, Nonablative or Laser Treatments, Nonablative or Nonablative Laser Treatments) 129530**

**#3 #1 and #2 55**

CKNI(34)

主题：激光＋激光治疗＋激光技术） AND （主题：肩峰下＋肩峰下撞击＋

肩峰下撞击综合征＋肩峰下滑囊＋肩峰下撞击征＋肩峰下撞击症） OR （主

题：肩峰下撞击＋肩峰下撞击综合征＋肩峰下撞击征＋肩峰下撞击症＋肩峰

下撞击综合症） OR （主题：肩峰下撞击综合征）

Wanfang(10)

主题：激光＋激光治疗＋激光技术） AND （主题：肩峰下＋肩峰下撞击＋

肩峰下撞击综合征＋肩峰下滑囊＋肩峰下撞击征＋肩峰下撞击症） OR （主

题：肩峰下撞击＋肩峰下撞击综合征＋肩峰下撞击征＋肩峰下撞击症＋肩峰

下撞击综合症） OR （主题：肩峰下撞击综合征）

CBM(5)

主题：激光＋激光治疗＋激光技术） AND （主题：肩峰下＋肩峰下撞击＋

肩峰下撞击综合征＋肩峰下滑囊＋肩峰下撞击征＋肩峰下撞击症） OR （主

题：肩峰下撞击＋肩峰下撞击综合征＋肩峰下撞击征＋肩峰下撞击症＋肩峰

下撞击综合症） OR （主题：肩峰下撞击综合征）

VIP(2)

主题：激光＋激光治疗＋激光技术） AND （主题：肩峰下＋肩峰下撞击＋

肩峰下撞击综合征＋肩峰下滑囊＋肩峰下撞击征＋肩峰下撞击症） OR （主

题：肩峰下撞击＋肩峰下撞击综合征＋肩峰下撞击征＋肩峰下撞击症＋肩峰

下撞击综合症） OR （主题：肩峰下撞击综合征）
