# Supplementary material for: Efficacy of low-level laser therapy for subacromial impingement syndrome: a meta-analysis of randomized controlled trials
Source: Lasers Med Sci. 2026 Sep 14;41(1):228. doi: 10.1007/s10103-026-05028-7 (PMC13572333; doi:10.1007/s10103-026-05028-7)
Supplement: Supplementary file 3 [file 10103_2026_5028_MOESM3_ESM.docx]

| **Question: VAS for [health problem] Bibliography:** . [Intervention] for [health problem]. Cochrane Database of Systematic Reviews [Year], Issue [Issue]. | | | | | | | | | | | |
| --- | --- | --- | --- | --- | --- | --- | --- | --- | --- | --- | --- |
| **Quality assessment** | | | | | | | **Summary of Findings** | | | | |
| **Participants (studies) Follow up** | **Risk of bias** | **Inconsistency** | **Indirectness** | **Imprecision** | **Publication bias** | **Overall quality of evidence** | **Study event rates (%)** | | **Relative effect** (95% CI) | **Anticipated absolute effects** | |
|  |  |  |  |  |  |  | **With Control** | **With VAS** |  | **Risk with Control** | **Risk difference with VAS** (95% CI) |
| **Shoulder pain** (Better indicated by lower values) | | | | | | | | | | | |
| 1090 (20 studies) | no serious risk of bias | very serious | no serious indirectness | no serious imprecision | undetected | ⊕⊕⊝⊝ **LOW** due to inconsistency | 564 | 526 | **-** |  | The mean shoulder pain in the intervention groups was **0.54 lower** (1.05 to 0.02 lower) |

**2.SPADI**

| **Question: SPADI for [health problem] Bibliography:** . [Intervention] for [health problem]. Cochrane Database of Systematic Reviews [Year], Issue [Issue]. | | | | | | | | | | | |
| --- | --- | --- | --- | --- | --- | --- | --- | --- | --- | --- | --- |
| **Quality assessment** | | | | | | | **Summary of Findings** | | | | |
| **Participants (studies) Follow up** | **Risk of bias** | **Inconsistency** | **Indirectness** | **Imprecision** | **Publication bias** | **Overall quality of evidence** | **Study event rates (%)** | | **Relative effect** (95% CI) | **Anticipated absolute effects** | |
|  |  |  |  |  |  |  | **With Control** | **With SPADI** |  | **Risk with Control** | **Risk difference with SPADI** (95% CI) |
| **Shoulder Pain and Disability** (Better indicated by lower values) | | | | | | | | | | | |
| 606 (12 studies) | no serious risk of bias | very serious | no serious indirectness | serious | undetected | ⊕⊝⊝⊝ **VERY LOW**^1^ due to inconsistency, imprecision | 315 | 291 | **-** |  | The mean shoulder pain and disability in the intervention groups was 3.61 **lower** (9.49 lower to 2.26 higher) |

**3.ROM**

| **Question: ROM for [health problem] Bibliography:** . [Intervention] for [health problem]. Cochrane Database of Systematic Reviews [Year], Issue [Issue]. | | | | | | | | | | | |
| --- | --- | --- | --- | --- | --- | --- | --- | --- | --- | --- | --- |
| **Quality assessment** | | | | | | | **Summary of Findings** | | | | |
| **Participants (studies) Follow up** | **Risk of bias** | **Inconsistency** | **Indirectness** | **Imprecision** | **Publication bias** | **Overall quality of evidence** | **Study event rates (%)** | | **Relative effect** (95% CI) | **Anticipated absolute effects** | |
|  |  |  |  |  |  |  | **With Control** | **With ROM** |  | **Risk with Control** | **Risk difference with ROM** (95% CI) |
| **Flexion ROM** (Better indicated by lower values) | | | | | | | | | | | |
| 744 (13 studies) | no serious risk of bias | very serious | no serious indirectness | no serious imprecision | undetected | ⊕⊕⊝⊝ **LOW**^1^ due to inconsistency | 383 | 361 | **-** |  | The mean flexion rom in the intervention groups was **4.35 higher** (1.13 to 7.58 higher) |
| **Extension ROM** (Better indicated by lower values) | | | | | | | | | | | |
| 340 (5 studies) | no serious risk of bias | very serious | no serious indirectness | serious | undetected | ⊕⊝⊝⊝ **VERY LOW**^1^ due to inconsistency, imprecision | 190 | 150 | **-** |  | The mean extension rom in the intervention groups was **0.79 higher** (0.47 lower to 2.06 higher) |
| **Abduction ROM** (Better indicated by lower values) | | | | | | | | | | | |
| 854 (15 studies) | no serious risk of bias | very serious | no serious indirectness | no serious imprecision | undetected | ⊕⊕⊝⊝ **LOW**^1^ due to inconsistency | 438 | 416 | **-** |  | The mean abduction rom in the intervention groups was **3.72 higher** (0.79 to 6.64 higher) |
| **Internal Rotation ROM** (Better indicated by lower values) | | | | | | | | | | | |
| 576 (10 studies) | no serious risk of bias | very serious | no serious indirectness | serious | undetected | ⊕⊝⊝⊝ **VERY LOW**^1^ due to inconsistency, imprecision | 299 | 277 | **-** |  | The mean internal rotation rom in the intervention groups was **1.14 higher** (1.51 lower to 3.78 higher) |
| **External Rotation ROM** (Better indicated by lower values) | | | | | | | | | | | |
| 794 (14 studies) | no serious risk of bias | very serious | no serious indirectness | no serious imprecision | undetected | ⊕⊕⊝⊝ **LOW** due to inconsistency | 408 | 386 | **-** |  | The mean external rotation rom in the intervention groups was **1.96 higher** (0.51 to 3.42 higher) |
