# Supplementary material for: Efficacy of low-level laser therapy for subacromial impingement syndrome: a meta-analysis of randomized controlled trials
Source: Lasers Med Sci. 2026 Sep 14;41(1):228. doi: 10.1007/s10103-026-05028-7 (PMC13572333; doi:10.1007/s10103-026-05028-7)
Supplement: Supplementary file 4 [file 10103_2026_5028_MOESM5_ESM.docx]

| **Outcome** | **Page** |
| --- | --- |
| **Pain** | **2-3** |
| **Shoulder Pain and Disability** | **4-5** |
| **Flexion** | **6-7** |
| **Abduction** | **8-9** |
| **External rotation** | **10-11** |
| **Internal rotation** | **12-13** |

**VAS**

1. **Egger’s test**

Number of studies = 20	Root MSE	=	2.906

------------------------------------------------------------------------------

Std_Eff | Coefficient Std. err. t	P>|t|	[95% conf. interval]

-------------+----------------------------------------------------------------

slope |	.3740634	.8659513	0.43	0.671	-1.445233	2.193359

bias | -2.567027	2.984368	-0.86	0.401	-8.836951	3.702897

------------------------------------------------------------------------------

Test of H0: no small-study effects	P = 0.401

2. **Funnel plots**

3. **Sensitivity analysis**

**SPADI**

1. **Egger’s test**

Number of studies = 12	Root MSE	=	2.128

------------------------------------------------------------------------------

Std_Eff | Coefficient Std. err. t	P>|t|	[95% conf. interval]

-------------+----------------------------------------------------------------

slope |	.1501908	.8521528	0.18	0.864	-1.748524	2.048906

bias | -1.245558	2.870783	-0.43	0.674	-7.64206	5.150945

------------------------------------------------------------------------------

Test of H0: no small-study effects	P = 0.674

**2.Funnel plots**

**3.Sensitivity analysis**

**ROM**

Flexion

1. **Egger’s test**

Number of studies = 13	Root MSE	=	4.425

------------------------------------------------------------------------------

Std_Eff | Coefficient Std. err. t	P>|t|	[95% conf. interval]

-------------+----------------------------------------------------------------

slope |	1.932521	1.635403	1.18	0.262	-1.666977	5.532018

bias |	1.070154	1.652409	0.65	0.531	-2.566774	4.707082

------------------------------------------------------------------------------

Test of H0: no small-study effects	P = 0.531

**2.Funnel plots**

**3.Sensitivity analysis**

Abduction

1. **Egger’s test**

Number of studies = 15	Root MSE	=	4.614

------------------------------------------------------------------------------

Std_Eff | Coefficient Std. err. t	P>|t|	[95% conf. interval]

-------------+----------------------------------------------------------------

slope |	.9984908	1.405405	0.71	0.490	-2.037702	4.034684

bias |	1.220811	1.564716	0.78	0.449	-2.159552	4.601174

------------------------------------------------------------------------------

Test of H0: no small-study effects	P = 0.449

**2.Funnel plots**

**3.Sensitivity analysis**

External rotation

1.**Egger’s test**

Number of studies = 14	Root MSE	=	2.442

------------------------------------------------------------------------------

Std_Eff | Coefficient Std. err. t	P>|t|	[95% conf. interval]

-------------+----------------------------------------------------------------

slope |	.4073982	.6241309	0.65	0.526	-.9524663	1.767263

bias |	.8400259	.8685841	0.97	0.353	-1.052456	2.732508

------------------------------------------------------------------------------

Test of H0: no small-study effects	P = 0.353

**2.Funnel plots**

**3.Sensitivity analysis**

Internal rotation

1.**Egger’s test**

Number of studies = 10	Root MSE	=	4.745

------------------------------------------------------------------------------

Std_Eff | Coefficient Std. err. t	P>|t|	[95% conf. interval]

-------------+----------------------------------------------------------------

slope |	1.763688	1.243495	1.42	0.194	-1.103816	4.631192

bias |	.1719599	1.979457	0.09	0.933	-4.392677	4.736597

------------------------------------------------------------------------------

Test of H0: no small-study effects	P = 0.933

**2.Funnel plots**

**3.Sensitivity analysis**
